# The Role of Immature Platelet Fraction and Reticulated Platelets in Stroke Monitoring and Outcome Prognosis: A Systematic Review

**DOI:** 10.3390/jcm14134760

**Published:** 2025-07-05

**Authors:** Alexandra Tsankof, Dimitrios A. Tsakiris, Lemonia Skoura, Panagiota Tsiatsiou, Eleftheria Ztriva, Georgios Ntaios, Christos Savopoulos, Georgia Kaiafa

**Affiliations:** 1 Intensive Care Unit, AHEPA University Hospital, Stilponos Kiriakidi 1, 54634 Thessaloniki, Greece; a.tsankof@gmail.com; 2 First Propaedeutic Department of Internal Medicine & Acute Stroke Unit, AHEPA University Hospital, Stilponos Kiriakidi 1, 54634 Thessaloniki, Greece; elztriva@gmail.com (E.Z.); chrisavopoulos@gmail.com (C.S.); gdkaiafa@yahoo.gr (G.K.); 3 School of Medicine, Faculty of Health Sciences, Aristotle University of Thessaloniki, University Campus, 54124 Thessaloniki, Greece; dtsakiris@icloud.com (D.A.T.); mollyskoura@gmail.com (L.S.); 4 Microbiology Department, AHEPA University Hospital, Stilponos Kiriakidi 1, 54634 Thessaloniki, Greece; ptsiatsiou@gmail.com

**Keywords:** immature platelet fraction, reticulated platelets, stroke, monitoring, outcome

## Abstract

**Background/Objectives**: Immature platelet fraction (IPF) and reticulated platelets (RPs) are biomarkers reflecting the youngest and most metabolically active platelets in circulation. RPs, a subset of immature platelets, contain residual RNA and have been associated with increased thrombotic potential. Elevated IPF levels indicate enhanced platelet production, commonly observed during elevated platelet turnover, such as in autoimmune reactions, consumption, and thrombotic events. This systematic review aims to evaluate the potential role of IPF and RPs in the context of cerebrovascular events, specifically ischemic and hemorrhagic stroke, as well as transient ischemic attacks (TIAs), and to assess their clinical utility in stroke monitoring and management. **Methods**: A comprehensive literature search was conducted in PubMed, Scopus, Cochrane Library, and Web of Science for studies published between 2000 and 2024, which focused on IPF and RPs in human subjects with cerebrovascular events. **Results**: Six studies met the inclusion criteria. Findings suggest that elevated levels of IPF and RP are associated with the acute and chronic phases of ischemic stroke and TIA and may reflect increased platelet turnover and thrombotic activity. Some evidence supports their role in predicting stroke severity, recurrence, and underlying etiology, although results are not yet consistent across all studies. **Conclusions**: IPF and RPs are emerging biomarkers with potential applications in acute ischemic stroke and risk stratification. While current evidence is promising, further research is needed to standardize measurement techniques and validate their routine use in clinical practice.

## 1. Introduction

Platelets play an essential role in the hemostatic response, contributing to the formation of a thrombus at sites of vascular injury, thereby participating in primary hemostasis, where their ability to adhere to the vessel wall and aggregate in response to injury is essential for maintaining vascular integrity [1,2]. The process is tightly regulated in a delicate balance, as excessive platelet activation can lead to thrombotic disorders, while insufficient activation can result in bleeding.

Platelet production or thrombopoiesis occurs in the bone marrow and is regulated by thrombopoietin (TPO), involving the maturation of megakaryocytes leading to the formation of proplatelets [3]. Platelet surface glycoproteins, including GPIa, GPIb/V/IX, and GPIIb/IIIa, play crucial roles in adhesion to collagen, von Willebrand factor (vWF), and fibrinogen, initiating thrombus formation [4,5].

Reticulated or immature platelets (RPs) arise from megakaryocyte fragmentation during thrombopoiesis and are characterized by their high RNA content. RPs remain in plasma for 24–36 h until transforming into the final mature platelet form. The presence of elevated RPs is a hallmark of accelerated platelet turnover, often observed in conditions associated with increased platelet destruction or consumption [6,7]. Given these functions, they are enzymatically and metabolically more active, which results in their enhanced prothrombotic potential [8,9]. When the demand for platelets is high, megakaryocytes release a larger amount of platelets into the circulation, seen as RPs in their early phase [10].

IPF represents the proportion of these immature platelets, and the normal reference range is 1.1–6.1% of the total platelet count [8]. IPF level measurement provides an estimate of platelet production from the bone marrow using a peripheral blood sample. IPF can be assessed using conventional flow cytometry or automated hematology analyzers [11]. While the terms RP and IPF are sometimes used interchangeably, they differ significantly in terms of measurement methodology and clinical availability, as shown in Table 1.

**Figure 1:**
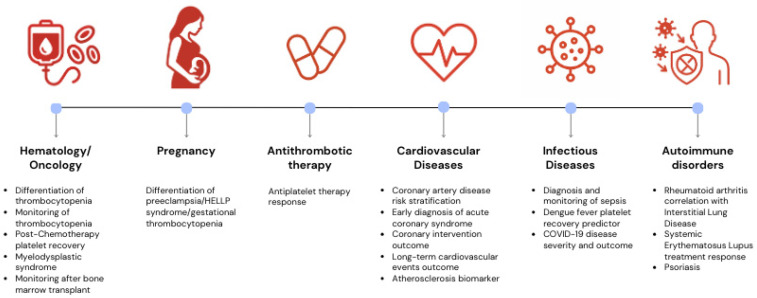
Current possible clinical applications of reticulated platelets and immature platelet fraction.

In recent years, IPF and RPs have garnered attention as potential biomarkers for assessing platelet dynamics in various cardiovascular conditions, as well as other conditions, reflecting their major influence in arterial micro- or macro-thrombotic events [12]. A synthesis of these potential conditions is shown in Figure 1.

Despite advances in stroke diagnostics and treatment, early risk stratification and monitoring of thrombotic activity remain clinical challenges. Traditional markers lack specificity for dynamic platelet turnover, limiting timely intervention. IPF and RP measurements offer a promising, non-invasive tool to fill this gap, potentially contributing to risk prediction, monitoring disease progression, and guiding antiplatelet therapy.

This systematic review aims to explore the role of IPF and RPs in the context of cerebrovascular events. While the literature on these biomarkers in stroke is still emerging, they are very promising as indicators of platelet dynamics, prognostic markers, and tools for stroke risk assessment. This review will examine the potential applications of IPF and RP in stroke monitoring and the clinical implications for their daily clinical application.

## 2. Materials and Methods

The protocol for our systematic review has been published and registered with PROSPERO (CRD420251050133). We reported the results of our search based on the Preferred Reporting Items for Systematic Reviews and Meta-Analysis (PRISMA) guidelines [13] (Supplementary Materials). Although this is a systematic review, we aimed to provide a synthesis of the current evidence by highlighting areas of consensus, discrepancies, and knowledge gaps in the literature. Risk of bias was assessed using an adapted version of the Newcastle–Ottawa Scale (NOS) tailored to each study design. No study was excluded based on risk of bias, but the variability in quality was considered during synthesis and interpretation.

### Study Selection

For this systematic review, a comprehensive literature search was conducted by two reviewers independently, in PubMed, Cochrane Library, Scopus, and Web of Science using the following search query: (“Stroke” OR “cerebrovascular event” OR “ischemic stroke” OR “transient ischemic attack”), AND (“Immature Platelet Fraction” OR “IPF” OR “Reticulated Platelets” OR “platelet activation” OR “platelet biomarkers”). We included original research articles, systematic reviews, and clinical studies that were published between 2000 and 2024, and focused on the role of IPF and RPs in cerebrovascular events, particularly in stroke. Studies were excluded if they were not published in English, lacked relevance to the topic (or primary focus on IPF and RPs), or were based on non-human models. Data were sought for the following outcomes: Diagnostic accuracy of IPF and RPs in stroke diagnosis, prognostic value predicting stroke recurrence, long-term functional outcomes such as National Institutes of Health Stroke Scale (NIHSS) and modified Rankin Scale (mRS), stroke severity (as assessed by the NIHSS or modified Rankin Scale), or stroke monitoring. All available data from included studies were sought for outcomes measured during the acute phase (within 72 h post-stroke) and chronic phase (up to 3 months post-stroke).

## 3. Results

### 3.1. Included Studies and Characteristics

**Figure 2:**
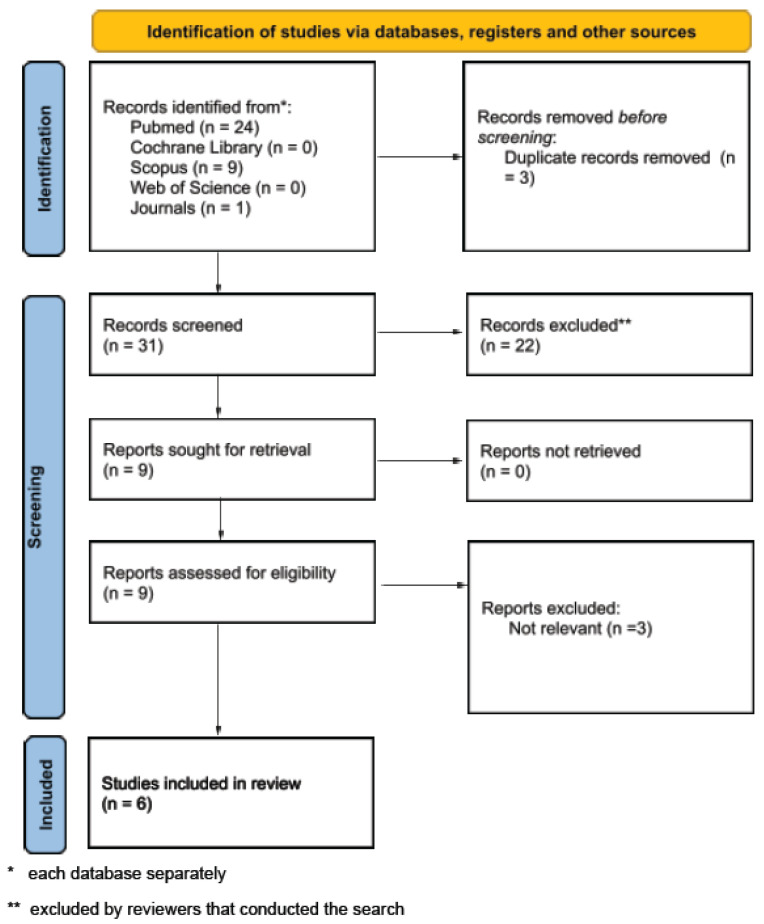
PRISMA 2020 flow diagram.

The study selection process is illustrated through the PRISMA flow diagram in Figure 2, while a PRISMA checklist was used to finalize the review form. The systematic literature search initially identified 34 potential studies, of which 3 were excluded due to duplication, and finally, 31 studies were screened. After the exclusion of 22 studies due to irrelevant study design or content, a total of 9 studies were retrieved. Eventually, six observational studies were included for review.

Specific study characteristics are analyzed in Table 2, based on year of publication (between 2002 and 2021), type of study (mainly observational studies either prospective or retrospective and longitudinal or cross-sectional), type of patients and grouping criteria (based on type of stroke or type of timing differentiation from the occurrence of stroke), and sample size (varying from 38 to 1655, with a median value 192).

Table 3 details the methodological approach used to quantify platelet indices, primarily through flow cytometry and automated hematology analyzers, with consistent use of nucleic acid staining and platelet-specific antibodies for accurate identification using various flow cytometry-based methodologies and hematology analyzers. Across the studies, RPs were measured via thiazole orange (TO) staining and platelet-specific antibodies such as cluster of differentiation 42b and 41 (CD42b, CD41), with quantification conducted on platforms such as the Coulter XL MCL, FACS Calibur, and Sysmex XE-2100 analyzers [14,15,16,17,18,19]. Platelet function assays were used in one study that evaluated the megakaryocyte–platelet–hemostatic axis [19]. Only one study referenced IPF measurement via automated hematology analyzers, which is more accessible in clinical settings [18]. Table 4 highlights the main results, antithrombotic regimens, and limitations extracted from each study.

### 3.2. Reticulated Platelets in the Acute and Chronic Phases of Stroke

Studies have shown that RPs play a crucial role in both acute and late phases of ischemic stroke and transient ischemic attack (TIA), reflecting ongoing platelet activation and turnover [14,15,16,17,18,19]. To our knowledge, this is one of the first studies to have shown a correlation of the percentage of reticulated platelets (%RP) to the early phase of stroke through flow cytometry, while comparing to the subacute (8–30 days) and late phases (>31 days) [16]. Another previous study underlined the upward trend of %RP in acute ischemic and hemorrhagic stroke while noting a shift in the megakaryocyte–platelet–hemostatic axis toward a more prothrombotic state in this early phase [19]. More recently, results from an investigation on the early neurological deterioration (END) of patients with acute stroke showed that a higher (>5%) IPF correlated with this category of clinical presentation of END in the acute phase [18]. Another study demonstrated that a significant increase in the percentage of circulating RPs (after age adjustment in the %RP) may be found during the early phase (up to 3 weeks) and late phase compared to controls following ischemic stroke or transient ischemic attack (TIA). The same study noted that, in the late phase (more than 3 weeks after stroke or TIA), a slight increase in the RP percentage could be found when adjusting for peripheral vascular disease [14].

On the other side, a cohort study also explored RP values across multiple time points (early, subacute, and late) after ischemic stroke and TIA [15]. Interestingly, a significant increase was found in RP percentage in the late phase (≥90 days) following TIA or ischemic stroke compared to healthy controls. In contrast, no significant increase in RP was found in the early or subacute phases (within 4 weeks) post-stroke [15]. This differs from other studies that reported elevated RP early after ischemic stroke, while it agrees with the results of patients when %RP is adjusted for peripheral vascular disease [14].

### 3.3. Association with Stroke Etiology

A study suggested that certain platelet characteristics, including RP levels, might be indicative of cardioembolic cause, a common etiology in patients with stroke [15]. Similarly, another observational study showed increased platelet count and RPs in patients with symptomatic carotid stenosis [17]. On the other hand, another study revealed, through a subgroup analysis based on the TOAST classification, a significantly higher %RP in patients with small vessel disease (SVD) or lacunar, both at baseline and 90 days, but no significant difference at 14 days [15]. In another study, no significant correlation was found between %RP and type of stroke [14].

### 3.4. Prognostic Value of RP/IPF in Stroke Outcome

A study found that RP levels remained elevated during the late phase and were predictive of poor prognosis, including possibly higher risks of major cardiovascular events [16]. Another study underlined the possible RP elevation in the late phase, after adjustment for peripheral vascular disease, indicating the connection with atherothrombotic risk [15].

## 4. Discussion

RPs have been shown to play a pivotal role in both the acute and chronic phases of ischemic stroke and TIA, acting as indicators of ongoing platelet activation and turnover. The early studies in this field demonstrated that RPs are significantly elevated in the acute phase of ischemic stroke. Other studies have further emphasized the increase in %RP in the acute phase of ischemic and hemorrhagic strokes, linking this to a shift in the megakaryocyte–platelet–hemostatic axis toward a more prothrombotic state [14,15,16,17,18,19]. This shift in platelet function during the acute phase suggests a heightened response to the ischemic insult, marked by increased platelet consumption and production at the injury site. Recent findings have also revealed that a higher IPF, specifically values greater than 5%, correlates with early neurological deterioration (END) in acute stroke patients. This further suggests that RP levels may not only reflect ongoing platelet turnover but could also be linked to the severity of clinical outcomes in the early phase of stroke [18]. Moreover, a study examining RP levels in the acute and late phases of ischemic stroke and transient ischemic attack (TIA) has highlighted significant findings. The study observed an increase in RP percentage in the early and late phases following ischemic stroke or TIA, after adjusting for age. This increase is likely a response to heightened platelet turnover and the release of younger, larger platelets into circulation as part of the body’s acute response to the ischemic injury. Interestingly, the same study found a slight increase in RP percentage in the late phase (beyond 3 weeks), which was adjusted for peripheral vascular disease. This suggests that, even after the acute event has stabilized, platelet turnover may remain elevated, particularly in patients with concurrent atherosclerotic diseases, signaling a prolonged recovery phase with continued platelet activity [14]. These findings suggest that the elevation of RPs during the early phase of ischemic stroke is not merely a pre-existing marker of stroke risk but likely serves as a biomarker of immediate response to the ischemic event. However, other studies have yielded different results, particularly regarding RP levels during the late phase of stroke. This observation differs from previous studies that demonstrated elevated RP levels in the early phase following ischemic stroke, but it aligns with findings where RP levels were adjusted for peripheral vascular disease, suggesting that vascular comorbidities might influence the trajectory of platelet turnover [14,15]. The discrepancy in findings between studies may stem from differences in methodology, such as the specific time point chosen for sampling or the patient populations studied, including the presence or absence of comorbidities like peripheral vascular disease. These variations suggest that RP elevation is more likely to be a reactive process following an ischemic cerebrovascular event, rather than a predictive marker for the onset of ischemic stroke or TIA.

Beyond their prognostic value regarding the acute and chronic phases of stroke, RPs have also been identified as potential markers for identifying the underlying etiology, particularly cardioembolic causes, which is one of the most common etiologies of ischemic stroke [16]. This notion aligns with previous research indicating that RP analysis can offer valuable insights into platelet activation patterns, which are integral in understanding the thrombotic mechanisms involved in stroke pathogenesis. Elevated RP levels in patients with cardioembolic stroke could point to heightened platelet activation, potentially contributing to the formation of thrombi that obstruct cerebral blood flow. On the other hand, a separate study that performed a subgroup analysis based on the TOAST classification revealed a significantly higher percentage of RPs in patients with small vessel disease (SVD) or lacunar infarctions, both at baseline and at 90 days post-stroke, with no notable difference observed at 14 days [15]. These findings suggest that SVD may have a distinct RP profile compared to other stroke subtypes. SVD is frequently associated with endothelial dysfunction, which impairs the function of the vascular endothelium, contributing to the promotion of platelet aggregation and thrombus formation. This endothelial damage leads to the release of prothrombotic factors, which play a crucial role in stimulating platelet activation, which in turn increases platelet turnover and may lead to the production of more RPs. In patients with ischemic stroke due to SVD, the combination of endothelial damage and sustained platelet activation results in a heightened state of platelet turnover, contributing to increased RP levels. Similarly, another observational study explored RP values in patients with carotid stenosis, a condition that often predisposes individuals to ischemic stroke. The study found increased platelet count and RP levels in these patients [17], suggesting that the heightened platelet activity in this cohort reflects an increased thrombotic risk. This is particularly relevant as carotid stenosis is frequently associated with microembolic events, which can further stimulate platelet activation. In this context, the study also focused on a cerebral microembolic signal (MES)-negative subgroup, which included patients who did not exhibit MES on transcranial Doppler (TCD) ultrasonography. The inclusion of this subgroup challenges the commonly held assumption that the presence of MES is directly correlated with stroke risk. MES, often observed in symptomatic carotid stenosis, is believed to result from microemboli, which can activate platelets and subsequently lead to increased production of RPs in response to heightened platelet turnover. An additional area warranting further investigation is the relationship between RP and IPF and carotid intima–media thickness (IMT), a validated marker of subclinical atherosclerosis and cardiovascular risk [20]. Such research could clarify the role of immature platelets in the early atherosclerotic process and potentially improve risk stratification in cerebrovascular disease.

This systematic review has several important limitations. Notably, only six studies fulfilled the predefined inclusion criteria, restricting the available evidence and underscoring the early stage of research on IPF and RP in cerebrovascular disease. The included studies varied widely across several fields. Given this considerable heterogeneity, conducting a meta-analysis was deemed inappropriate, as combining such diverse data would not demonstrate reliable or clinically meaningful conclusions. Consequently, a structured qualitative synthesis was chosen to preserve the validity of the findings. Future investigations with larger cohorts, standardized methodologies, and consistent outcome reporting are necessary to enable meaningful meta-analyses and clarify the clinical utility of IPF and RP in stroke diagnosis and prognosis.

One limitation common to several of these studies is the variability in sample size. For instance, some studies included relatively small sample sizes, which can limit the applicability of the findings to broader populations. Large sample sizes in certain studies (such as those including over 1000 participants) can introduce a selection bias if the included population does not adequately represent the full spectrum of stroke subtypes, comorbidities, or demographic characteristics. Another significant limitation stems from the methodological variability in measuring RP levels across studies. Different studies employed varying laboratory techniques, including flow cytometry and other platelet activation assays, to assess RP percentages. This lack of standardization in measurement methods could result in inconsistencies in RP quantification, making it difficult to directly compare findings across studies. The timing of sample collection in relation to the stroke event represents another limitation. Several studies assessed RP levels at varying time points across the acute, subacute, and chronic phases of stroke, but the precise timing of sampling can significantly influence RP levels. Early-phase studies (within 24 to 72 h post-stroke) may reflect an acute inflammatory response with elevated platelet turnover, whereas later-phase studies (90 days post-stroke) may capture changes in platelet function that are influenced by recovery and stabilization processes.

Furthermore, many studies reviewed did not adequately control potential underlying comorbidities or the use of medication that may influence platelet function and turnover. Peripheral vascular disease and other forms of atherosclerosis, which were found to influence RP levels in some studies, could confound the relationship between RP levels and stroke outcome. Many stroke patients are prescribed medications such as aspirin or P2Y12 inhibitors either acutely or as part of secondary prevention. These therapies alter platelet function and may provoke compensatory shifts in platelet production, potentially leading to changes in RP and IPF levels independent of the underlying stroke pathology. The inconsistent reporting of antiplatelet therapy across studies represents a significant limitation in the current literature, as it can obscure genuine relationships between platelet turnover markers and clinical outcomes. Notably, there was variability in treatment strategies among patient groups, including a small proportion without any antiplatelet therapy, especially within asymptomatic or control cohorts [Table 4]. These findings highlight the critical need for standardized and detailed reporting of antithrombotic therapy in future studies to enable accurate interpretation of IPF and RP levels and their clinical significance in cerebrovascular disease.

Another limitation common to many studies is the lack of long-term follow-up. While some studies reported RP levels up to 90 days post-stroke, few studies have tracked RP levels beyond this period. Long-term monitoring of RP levels would provide further insights into the persistence of elevated platelet turnover after the acute phase and its potential relationship with long-term stroke recovery, recurrent events, or other vascular outcomes.

Regarding the potential clinical applications of RP and IPF, the growing body of evidence suggests that measuring RP levels can be studied in order to serve as a non-invasive tool for monitoring platelet dynamics after ischemic stroke and TIA, especially on the basis of the chronologically early studies mentioned before. Therefore, incorporating RPs and IPF into etiology assessment, such as carotid disease, could enhance risk stratification by identifying patients with active platelet turnover who may benefit from closer monitoring or intensified antithrombotic therapy. Furthermore, IPF measurement offers several advantages as a clinical tool, including its low cost, speed of measurement using automated blood analyzers, and ease of incorporation into routine clinical practice. Current methods for measuring IPF, such as automated flow cytometry-based technologies, allow for rapid and accurate determination of platelet turnover, making it an appealing biomarker in acute clinical settings. However, challenges remain in terms of standardization of IPF measurements, the interpretation of IPF results in different clinical contexts, and the integration of IPF with other biomarkers or clinical findings to improve patient outcomes. Despite these challenges, IPF is an emerging biomarker in stroke management, particularly for monitoring platelet dynamics and guiding therapeutic strategies. As research continues, the clinical utility of IPF may expand to include risk stratification, early detection of thrombotic events, and personalized treatment plans for patients with ischemic stroke and TIA.

## 5. Conclusions

IPF and RPs have attracted interest as potential monitoring markers in the context of stroke, particularly in understanding platelet dynamics and their role in stroke pathophysiology. Elevated IPF/RP levels during the acute and late phases of stroke reflect increased platelet production and thrombotic activity, serving as an indicator of disease severity and prognosis. Furthermore, IPF/RP measurements may help predict stroke recurrence and assess long-term cardiovascular risk. Despite some challenges in its clinical application, IPF offers a rapid, cost-effective tool for monitoring platelet dynamics, with the potential to improve stroke management and patient outcomes. Further studies are needed to standardize its use and fully understand its role in clinical practice and to validate its use in guiding therapeutic decisions in stroke care.

## Figures and Tables

**Table 1:** Comparative overview of immature platelet fraction (IPF) and reticulated platelets (RPs).

Aspect	Reticulated Platelets	Immature Platelet Fraction
Definition	Subset of young platelets	Percentage of immature platelets to total platelet count
Measuring method	Manual or semi-automated flow cytometry	Fully automated hematology analyzers
Staining	Thiazole orange (or any other RNA-binding dye) combined with CD41/42b for platelet gating	RNA-sensitive fluorescent dye (analyzer reagent)
Specificity	High	High
Availability	Usually limited to research centers	Widely available
Analysis time	1–2 h	Rapid (within minutes)
Limitations	Requires expertise, lack of standardization, and sample aging affects results	Lower resolution for identifying platelet subtypes, dependent on proprietary algorithms

CD42b: cluster of differentiation 42b; CD41: cluster of differentiation 41; RNA: ribonucleic acid.

**Table 2:** Basic study characteristics.

Study	Year	Type of Study	Sample Size	Type of Stroke	Grouping Criteria
McCabe et al. [14]	2004	Observational	176	Ischemic or TIA	Early phase (1–27 days, n = 79), Late phase (79–725 days, n = 70), and Control (n = 27)
Lim et al. [15]	2020	Observational	244	Ischemic or TIA	Early (<4 weeks, n = 210), with follow up (n = 182), Late (>90 days, n = 145), and Control group (n = 34)
Nakamura et al. [16]	2002	Observational	208	Ischemic	Acute (1–7 days, n = 10), Subacute (8–30 days, n = 12), Chronic (>31 days, n = 46) and Control (n = 140)
Murphy et al. [17]	2018	Observational	114	LLA type or TIA	Early (n = 43), Late (n = 37) and Asymptomatic (n = 34)
Cho et al. [18]	2021	Observational	16,551	Ischemic	Subgroup (n = 72, 4.4%) from a stroke registry of 1655 patients
Smith et al. [19]	2002	Observational	38	Ischemic or Hemorrhagic	Acute (n = 24) and Control group (n = 14)

TIA: transient ischemic attack; LAA: large-artery atherosclerosis.

**Table 3:** Overview of methodological techniques in reviewed studies.

Study	Methodology
McCabe et al. [14]	Sysmex XE-2100 Hematology Analyzer and Coulter EPICS XL-MCL flow cytometer (with thiazole orange staining and CD42b antibody)
Lim et al. [15]	Sysmex XE-2100 Hematology Analyzer and Beckman Coulter XL MCL flow cytometer (with thiazole orange and CD42b antibody conjugated to PE)
Nakamura et al. [16]	Coulter Profile II flow cytometer (with thiazole orange for CD41 antibody)
Murphy et al. [17]	Sysmex XE-2100 Hematology Analyzer and Beckman Coulter flow cytometer (with thiazole orange and CD42b antibody)
Cho et al. [18]	Sysmex XE-2100 Hematology Analyzer
Smith et al. [19]	Sysmex series 9000 Hematology Analyzer

CD42b: Cluster of Differentiation 42b; CD41: Cluster of Differentiation 41; PE: Phycoerythrin.

**Table 4:** Overview of study outcomes, antithrombotic therapy, and limitations.

Study	Outcomes	Antithrombotic Therapy	Limitations
McCabe et al. [14]	No significant increase in unadjusted %RP in early or late-phase stroke/TIA patients compared to controls. (only after age and PVD adjustment). No significant effect of aspirin dose escalation	Aspirin monotherapy (early: 70%, late: 64%). Some on combinations (Aspirin + dipyridamole or clopidogrel) Controls untreated (85%).	Small sample size in the aspirin escalation group.
Lim et al. [15]	%RP was significantly increased in the late phase. SVD had a significantly higher %RP at baseline and 90 days	Dynamic regimens over time: Baseline: 76% on aspirin monotherapy, 22% no antiplatelet 14 days: 47% on aspirin + dipyridamole, 34% clopidogrel, 19% aspirin alone 90 days: 52% Aspirin + dipyridamole, 33% clopidogrel, 15% aspirin alone	Selection bias due to the two included studies. Variations of inclusion time for the acute phase.
Nakamura et al. [16]	%RP was significantly higher in patients with cardioembolic stroke and lower in patients under antithrombotic treatments	No antithrombotic (n = 8) Antiplatelet therapy (n = 50) Anticoagulant therapy (n = 16)	Small sample size to correlate with stroke phases and treatment response
Murphy et al. [17]	Automated %RPF and #RP were significantly higher in early symptomatic than asymptomatic MES−ve patients. No significant differences in patients on specific antiplatelet regimens	Aspirin monotherapy (64.7%) Combination therapies No antiplatelet therapy (Asymptomatic, 2.9%). Median daily aspirin dose elevated in early symptomatic patients	Small sample size for recurrent event prediction or event prognosis.
Cho et al. [18]	High IPF was an independent predictor of the prevalence of END.	No mention of antithrombotic therapy	Single-center study with retrospective design. Low prevalence of END may limit statistical power.
Smith et al. [19]	A trend towards an increase in %RP was found in stroke patients	No mention of antithrombotic therapy	Small sample size with incomplete matching of all vascular risk factors.

%RP: percentage of reticulated platelets among total platelet count; #RP: absolute reticulated platelet count; TIA: transient ischemic attack; PVD: peripheral vascular disease; SVD: small vessel disease; MES-ve: microembolic signal negative; END: early neurological deterioration.

## Supplementary Materials

The following supporting information can be downloaded at: https://www.mdpi.com/article/10.3390/jcm14134760/s1, Table S1: PRISMA 2020 Abstract Checklist; Table S2: PRISMA 2020 Checklist [13].
